# Differences in the transcriptome of medullary thyroid cancer regarding the status and type of *RET* gene mutations

**DOI:** 10.1038/srep42074

**Published:** 2017-02-09

**Authors:** Malgorzata Oczko-Wojciechowska, Michal Swierniak, Jolanta Krajewska, Malgorzata Kowalska, Monika Kowal, Tomasz Stokowy, Bartosz Wojtas, Dagmara Rusinek, Agnieszka Pawlaczek, Agnieszka Czarniecka, Sylwia Szpak-Ulczok, Tomasz Gawlik, Ewa Chmielik, Tomasz Tyszkiewicz, Barbara Nikiel, Dariusz Lange, Michal Jarzab, Malgorzata Wiench, Barbara Jarzab

**Affiliations:** 1 Department of Nuclear Medicine and Endocrine Oncology, Maria Sklodowska-Curie Memorial Cancer Center and Institute of Oncology, Gliwice Branch, Poland; 2 Genomic Medicine, Medical University of Warsaw, Warsaw, Poland; 3 Department of Clinical Science, University of Bergen, Bergen, Norway; 4 The Oncology and Reconstructive Surgery Clinic, Maria Sklodowska-Curie Memorial Cancer Center and Institute of Oncology, Gliwice Branch, Gliwice, Poland; 5 Tumour Pathology Department, Maria Sklodowska-Curie Memorial Cancer Center and Institute of Oncology, Gliwice Branch, Gliwice, Poland; 6 III Radiotherapy Clinic, Maria Sklodowska-Curie Memorial Cancer Center and Institute of Oncology, Gliwice Branch, Gliwice, Poland; 7 College of Medical and Dental Sciences, University of Birmingham, Birmingham, United Kingdom

## Abstract

Medullary thyroid cancer (MTC) can be caused by germline mutations of the *RET* proto-oncogene or occurs as a sporadic form. It is well known that *RET* mutations affecting the cysteine-rich region of the protein (MEN2A-like mutations) are correlated with different phenotypes than those in the kinase domain (MEN2B-like mutations). Our aim was to analyse the whole-gene expression profile of MTC with regard to the type of *RET* gene mutation and the cancer genetic background (hereditary vs sporadic). We studied 86 MTC samples. We demonstrated that there were no distinct differences in the gene expression profiles of hereditary and sporadic MTCs. This suggests a homogeneous nature of MTC. We also noticed that the site of the *RET* gene mutation slightly influenced the gene expression profile of MTC. We found a significant association between the localization of *RET* mutations and the expression of three genes: *NNAT* (suggested to be a tumour suppressor gene), *CDC14B* (involved in cell cycle control) and *NTRK3* (tyrosine receptor kinase that undergoes rearrangement in papillary thyroid cancer). This study suggests that these genes are significantly deregulated in tumours with MEN2A-like and MEN2B-like mutations; however, further investigations are necessary to demonstrate any clinical impact of these findings.

Medullary thyroid cancer (MTC) is currently a useful model for cancer therapy because its strong genetic predisposition provides a rationale for clinical management based on patient genotype. Moreover, following the identification of the tyrosine kinase activity of the RET protein, targeted therapy of MTC has been introduced recently into clinical use, providing an option for patients with advanced disease. Both these issues raise questions about the molecular events, which drive the phenotype of MTC. MTC arises from parafollicular C cells of the thyroid and accounts for a relatively low proportion (3–5%) of all thyroid cancers^1^,^2^,^3^. However, the estimated number of MTC cases worldwide is high since the incidence ranges from 0.11 to 0.21 per 100,000 person/year^4^. MTC occurs as both a hereditary disease (20–30% of all cases) and a sporadic disease^5^,^6^. The hereditary type is a consequence of germline mutations in the *RET* proto-oncogene and is associated with multiple endocrine neoplasia type 2 syndrome (MEN2), inherited in an autosomal dominant pattern. Historically, MEN2 syndrome has been classified into three distinct variants: MEN2A (OMIM 171400), which occurs in 80% of all hereditary cases^7^,^8^, familial medullary thyroid cancer (FMTC) (OMIM 155240), arising in up to 57% of all hereditary cases, and MEN2B (OMIM 162300), in approximately 5% of all hereditary MTCs^9^. Normally, ligand binding triggers the dimerization and activation of the RET receptor, which induces a signal transduction pathway leading to cell proliferation^10^,^11^. In the absence of a ligand, the RET protein is a single unphosphorylated tyrosine kinase receptor, but in cancer cells, single point mutations in the *RET* proto-oncogene lead to the autophosphorylation of tyrosine residues, which consequently induces the constitutive activation of the RET receptor and a permanent gain-of-function^12^,^13^. There is a significant relationship between the site of *RET* mutation and the phenotype, as well as the clinical course of MEN2 syndrome, and it is believed that *RET* mutations exhibit different transforming potentials depending on their location within the RET molecule^7^,^8^,^14^,^15^.

The RET protein is composed of three domains. The extracellular domain includes a ligand binding site, a cadherin-like domain, and a cysteine-rich domain. Mutations located in the extracellular domain may activate the ligand-independent dimerization and autophosphorylation of the RET protein. Mutations located in this region are mainly associated with MEN2A and more rarely with FMTC^12^,^16^. The most frequent *RET* mutations affecting codon 634 (exon 11) are detectable in up to 85% of all MEN2A patients, while mutations in codons 609, 611, 618 and 620 (exon 10) account for 10–15%^2^,^17^,^18^. Mutations in codon 10 are also observed in FMTC syndrome, in which MTC is the only symptom of the disease, often with a late onset and a more benign clinical course. However, the distinction between FMTC and MEN2A is rather debatable, and FMTC is often considered as a phenotypic variant of MEN2A^19^,^20^. The second RET protein domain is a hydrophobic region located within the cell membrane, and the third one is an intracellular tyrosine kinase domain (TK), which contains two tyrosine residues (TK1 and TK2). Mutations in this domain (mainly in codon 918 of the TK2 domain) lead to the autophosphorylation of the protein and activate the RET receptor without dimerization, but there is a distinct difference in the transforming activity among those mutations. Mutations affecting the first kinase subdomain (TK1), related to exons 13 and 14, are mainly observed in FMTC syndrome, whereas those involving the second kinase subdomain (TK2) are usually observed in codon 918. Germline mutations are associated with the most aggressive presentation of MTC, MEN2B syndrome, which is characterized by an earlier age of onset, developmental abnormalities including marfanoid habitus with skeletal deformations, intestinal and mucosal ganglioneuromatosis, and pheochromocytoma in 50% of cases. It has been suggested that the activation of the RET receptor due to a germline *RET* 918 mutation is related to an alteration in the substrate recognition pocket of the catalytic core and facilitates the phosphorylation of novel substrates^13^,^17^,21, which may lead to the induction of different downstream pathways. Seventy five percent of MTC patients have no familial history. Somatic *RET* mutations are observed in 40–70% of sporadic tumours^22^,^23^,^24^,^25^. Additionally, *RAS* mutations are detected in a high proportion of *RET*-negative tumours^26^,^27^; however, the role of somatic *RAS* mutations in MTC development is still not clearly demonstrated. As MEN2B-like mutations are considered the major somatic events in sporadic cancer, o a paradoxical discrepancy occurs between the possibly more aggressive clinical course of somatic *RET* 918-induced cancer and the generally better prognoses in all sporadic cancers.

It would, therefore, be valuable to compare gene expression profiles not only between hereditary and sporadic MTC but also between sporadic MTC caused by MEN2B-like mutations in *RET* exon 16 and MEN2A-like mutations in codons 10–11. Only a few similar comparisons have been reported, and the results are ambiguous due to the small number of tumours analysed^28^,^29^,^30^,^31^. In our study, two null hypotheses were tested:

1. There is no difference in the gene expression profiles of hereditary and sporadic MTC.
2. There is no difference in the gene expression profiles of MTC caused by MEN2A-like or MEN2B-like mutations.

For the purpose of this study, all mutations in *RET* exons 10 and 11, both hereditary and sporadic, were classified as MEN2A-like, whereas both germline and somatic mutations in *RET* exon 16 were classified as MEN2B-like.

## Results

### Differences in the gene expression profiles of hereditary and sporadic MTC

**Figure 1:**
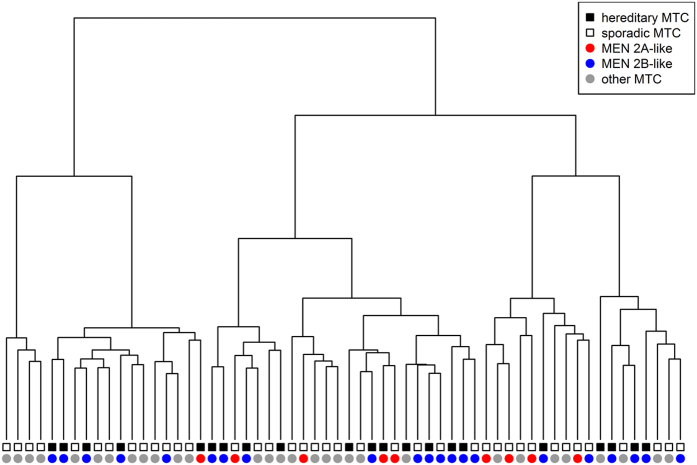
Hierarchical clustering of MTC samples based on microarray results.

Differences in the gene expression profiles were analysed in a dataset of 60 MTCs including 22 hereditary and 38 sporadic cases (Set A) (Supplementary Information Table S1). Using unsupervised analysis by hierarchical clustering, we did not observe global differences in the gene expression profiles between patients in whom the disease was caused by germline *RET* mutation and those with sporadic MTC, either with or without somatic *RET* mutation (Fig. 1). We then carried out a supervised analysis with 15 probe sets, which detected differential expression between the subtypes (FDR <0.05). The results are summarized in Table 1.

### Genes differing in expression between MTC tumours caused by different types of *RET* mutations

The gene expression profiles were compared for 21 MEN2A-like samples (5 somatic and 16 hereditary) and 9 MEN2B-like samples (7 somatic and 2 hereditary) (Set B) (Supplementary Information Table S1). While hierarchical clustering did not indicate any global influence of the type of *RET* mutation on the gene expression profile (Fig. 1), further analysis using a supervised method revealed ten genes for which the expression was significantly changed (FDR <0.05) in tumours with MEN2A-like mutations compared to tumours with MEN2B-like mutations (Table 2).

### *NTRK3* expression and *RET* mutation type

**Figure 2:**
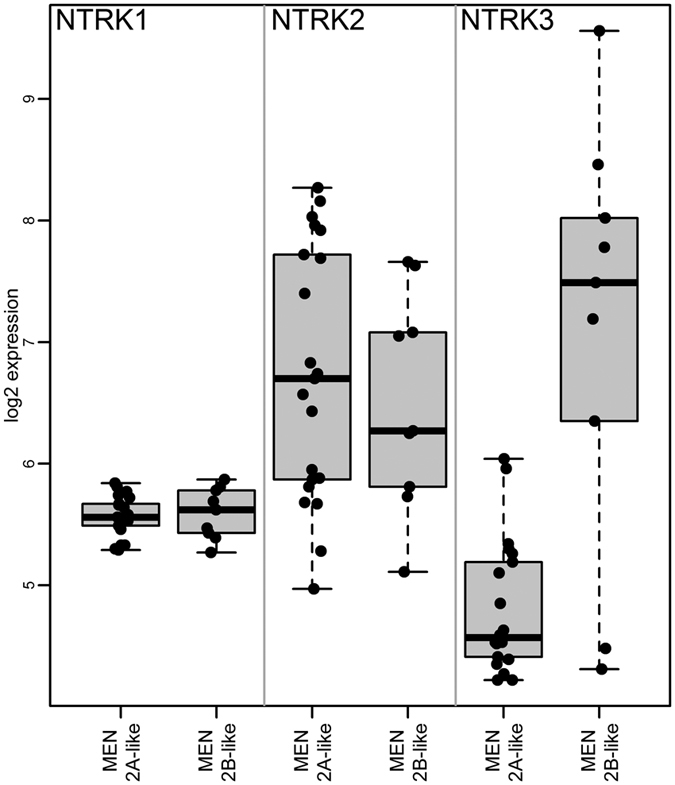
Expression of 3 types of NTRK receptors in MEN2A-like and MEN 2B-like samples in the microarray analysis.

In the microarray supervised data analysis, *NTRK3* was the most significantly deregulated gene, almost five times higher in MEN2B-like samples than in MEN2A-like samples. For comparison, the two other variants of the NTRK receptor (*NTRK1, NTRK2*) were also examined in our transcriptome profiling, but no association with *RET* mutation type was observed for either of them (*NTRK1* was filtered out during the pre-processing step, and *NTRK2* was insignificant (FDR = 0.86) (Fig. 2). Twenty-four samples were checked according to the presence of the ETS variant 6 gene (*ETV6*)-neurotropin receptor 3 (*NTRK3*) rearrangement (ETV6-NTRK3). The ETV6-NTRK3 fusion gene was not detected in any of the analysed MTC samples (Supplementary Information Fig. S2).

### Tumour classification by *RET* mutation type

To verify how the observed differences delineated the MTC samples with different *RET* mutation subtypes, we trained a 4-gene classifier (Table 3). Within this classifier, 2 genes (*NNAT* and *PTPRT*) were up-regulated for MEN2A-like mutations, and 2 genes (*NTRK3* and *GABRR1*) were up-regulated for MEN2B-like mutations. LOOCV resulted in the correct classification of 20/21 *RET* MEN2A-like mutation samples and 7/9 *RET* MEN2B-like mutations, thus showing an accuracy of 90%, a sensitivity of 91%, and a specificity of 87.5% (Table 4).

### Validation of the microarray data using independent sets of samples

#### Hereditary MTC vs sporadic MTC

We validated genes found as significantly deregulated between hereditary and sporadic MTCs, based on the microarray set (Affymetrix Gene 1.0 ST), on independent MTC samples analysed in our previous study^32^. Among 15 genes, 8 were found to be insignificant. The other 7 genes were not represented on the type of microarrays used in previous experiment (Affymetrix HG-U 133 A). We assessed those genes by RT-qPCR validation and did not see significant changes in expression between sporadic and hereditary forms of MTC.

#### MEN2A-like vs MEN2B-like mutations

**Figure 3:**
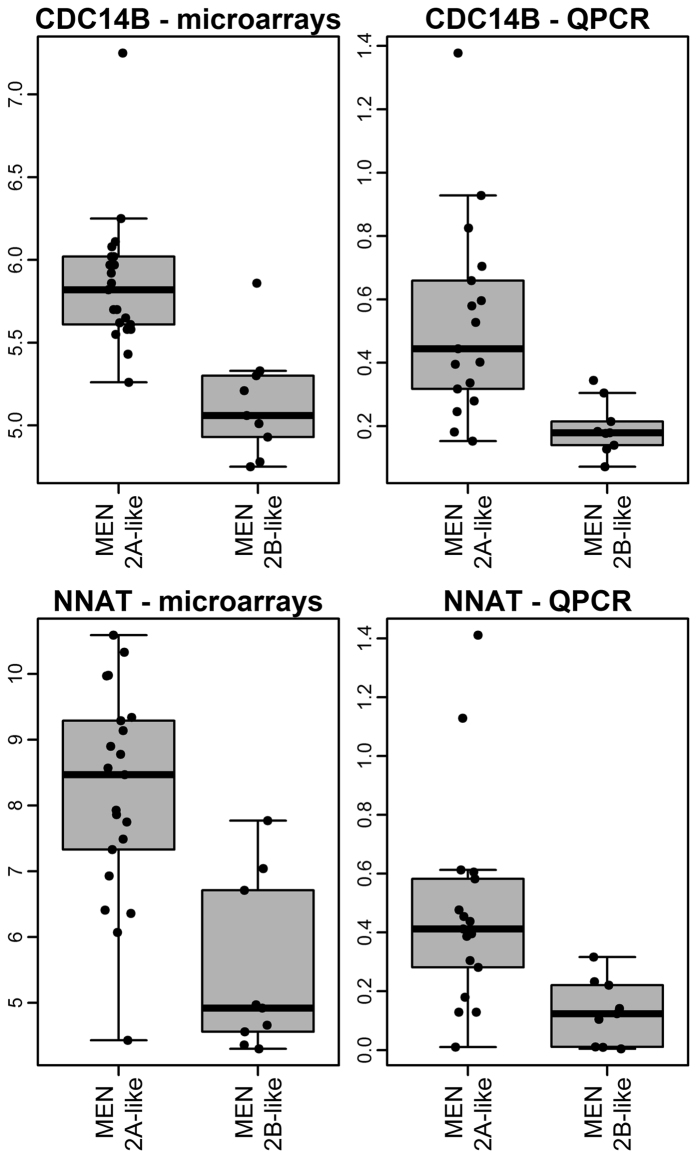
Expression of *CDC14B* and *NNAT* genes in the microarray analysis and the RT-qPCR validation of independent sets of samples.

For validation related to differences in the gene expression profiles dependent on *RET* mutation status, we applied the RT-qPCR method. For this purpose, we used 25 additional MTC samples, 16 with MEN2A-like mutations and 9 with MEN2B-like mutations. From 10 genes that were significantly differentially expressed (FDR <0.05), we were able to analyse 9 because, due to the difficulties with the insufficient quality of amplification, we had to exclude 1 gene (Supplementary Information Table S2). Using the validation set, we confirmed the overexpression of *NNAT* (Bonferroni adjusted p-value = 0.01, fold change = 3.3) and *CDC14B* (Bonferroni adjusted p-value = 0.003, fold change = 2.8) genes in samples with MEN2A-like mutations (p < 0,001) (Fig. 3). The expression of the other genes analysed did not show significant differences related to the *RET* mutation type.

Despite using two independent amplicon designs and methodologies (Roche Diagnostic GmbH, Mannheim, Germany and TaqMan MGB, Life Technologies, Carlsbad, USA), we were also not able to detect significant differences in the microarray part of the study for *NTRK3* expression. We observed very low amplification of the standard curve, and the samples resulted in very low efficiency of amplicons, suggesting technical difficulties with the assay. Therefore, we attempted the validation of the results for *NTRK3* using immunohistochemistry.

### Immunohistochemistry of the protein product of the *NTRK3* gene

We analysed 12 samples: 5 MEN2A-like and 7 MEN2B-like samples. We observed very strong (+++) and strong (++) immunohistochemical staining with anti-TrkC antibody in all MEN2B-like cases. TrkC was localized mainly in the cytoplasm and in the nuclei of tumour cells in 3 cases. Immunostaining revealed that in these slides, TrkC was also present in follicular thyroid cells. On the other hand, all but one MEN 2A-like tumour were negative in TrkC staining for both tumour and follicular cells. Only one sample showed a positive reaction (Supplementary Information, Fig. S1).

## Discussion

Differences in the gene expression profiles are usually easily observed when tumour tissue is compared to the corresponding normal tissue or between tumours of different genetic backgrounds (e.g., medullary versus papillary thyroid carcinoma). However, the identification of reliable gene expression biomarkers for tumours with more subtle differences, like distinct founding gene mutations, especially when affecting the same gene, is more difficult and requires much larger datasets to obtain consistent and reproducible results^33^, and such datasets are often difficult to collect when relatively rare conditions are studied. In MTC, a number of subsets must be considered, dependent on whether the disease is hereditary or sporadic, further complicated by the fact that each of these could be caused by gene mutations in the extracellular or intracellular domain of the RET protein or might not demonstrate *RET* mutations. Thus far, there have been only a few reports published that are dedicated to MTC, most of which represent limited datasets (23, 13 and 41 MTC cases)^28^,^30^,^31^. Here, we present the results of gene expression profiling in which all MTC subsets are well represented.

Our results clearly demonstrate that there were no distinct differences in the gene expression profiles of hereditary and sporadic MTCs. Therefore, we found a rather homogeneous nature with respect to MTC. Although we found 15 differentially expressed genes characterized by an FDR of <0.05 on microarrays, none of the 14 validated genes were significantly deregulated when tested by an independent group of samples.

Ameur *et al*.^30^ suggested that similar pathways might be activated in both sporadic and hereditary MTC and further linked the differences in expression of the genes with the aggressiveness of the tumour. However, they analysed a limited set of samples (4 hereditary MTC and 7 sporadic MTC)^30^. Conversely, Maliszewska *et al*.^31^ argued that familial and sporadic MTC cases are characterized by differential gene expression patterns, but this analysis considered almost all familial cases with a mutation in *RET* codon 634 without any sporadic cases^31^.

Similarly, in the case of breast cancer, a landmark genomic study suggested that hereditary and sporadic breast cancers are clearly distinguishable^33^, but we and others were able to demonstrate subsequently that other sources of heterogeneity might have influenced these conclusions and that hereditary and sporadic breast carcinomas are not in fact dissimilar^34^,^35^,^36^. Differences between hereditary and sporadic cancers may also be attributed to epigenetic events, for example, microRNA silencing^37^, and their role will probably be further investigated in the future.

Due to the strong genotype-phenotype correlation of the type of *RET* gene mutation and the clinical outcome, it has been proposed that *RET* mutations in MEN2A and MEN2B tumours result in changes of distinctive gene expression profiles and signalling pathways^28^,^29^,^31^. We identified 10 genes (FDR <0.05) by microarrays, whose expression distinguished MTC tumours with MEN2A-like mutations from those with MEN2B-like mutations. Among 9 genes validated by RT-qPCR on an independent set of samples, the differences were confirmed for only *NNAT* and *CDC14B*, while the other genes were not positively validated by the RT-qPCR.

We observed the highest fold change for the *NNAT* (neuronatin) gene among all differentially expressed genes, according to the *RET* mutation type. It is a brain-specific gene with high expression during embryogenesis and is normally silenced in differentiated neurons^38^. The overexpression of neuronatin is associated with cell proliferation and the shorter survival of glioblastoma patients^39^ and also promotes medulloblastoma growth^40^. In our data, the overexpression of the *NNAT* gene was observed in tumours with MEN2A-like mutations. Most of our MEN2A-like samples harbour a *RET*634 mutation, a type of mutation which correlates with poor prognoses among all MEN2A-like mutations. Mise *et al*. verified the high proliferation activity of tumours with MEN2A-like mutations by a transforming assay performed in NIH3T3 fibroblasts and an *in vivo* test in nude mice^14^. Mise *et al*. suggested that MEN2A mutations accelerate cell proliferation, while MEN2B mutations suppressed apoptosis^14^. They observed that cells with a RETC634R mutation, characteristic for MEN2A, grew rapidly. *In vitro* tests confirmed the results as the tumour growth was substantially faster in mice injected with NIH3T3 cells transfected with MEN2A-like mutation^14^. Interestingly, *NNAT* was also included in the list of genes up-regulated in MEN2A samples by Jain *et al*.^28^, but it was insignificant (FDR >0.1).

The second gene, *CDC14B* (cell division cycle 14B), was also over-expressed in MEN2A-like samples. This gene is a dual-specificity phosphatase that regulates the cell cycle^41^. The role in the tumour transformation ability of *CDC14B* gene is controversial. There are studies suggesting that the overexpression of the *CDC14B* gene induced the oncogenic transformation of transfected NIH3T3 cells and altered their motility^42^. On the other hand, there are some studies showing the down-regulation or loss of *CDC14B* expression in many tumours arising from breast, prostate, ovary, liver and brain^43^ and the inhibition of glioblastoma growth^44^. The down-regulation of *CDC14B* also correlated with tumour recurrence and shorter recurrence-free survival in renal cell carcinoma^45^. In our study, the down-regulated expression of *CDC14B* was observed in MEN2B-like mutation samples and believed to be clinically more aggressive. Additionally, the somatic M918T mutation correlates with worse MTC outcomes^22^. These findings might suggest that the expression of *CDC14B* modulates the prognosis in medullary thyroid cancer.

In our microarray studies, we observed the most significant difference in gene expression between MEN2B-like and MEN2A-like *RET* mutations, regarding the *NTRK3* gene. This tyrosine kinase receptor gene, important for nerve growth factor signal transduction, plays a significant role in neuroendocrine carcinomas^46^,^47^. The overexpression of *NTRK3* could be associated with the potentially higher clinical aggressiveness of MTC induced by MEN2B-like mutations. This observation is consistent with the results of a McGregor *et al*. study, where increased strong immunostaining of NTRK3 protein was noted in 87% of 25 analysed MTC samples. The authors correlated *NTRK3* expression with aggressive metastatic tumours^48^, but that study did not consider the *RET* mutation status. Expression of TrkC promotes breast tumour growth and metastasis^49^. High expression of *NTRK3* was observed in our MEN2B-like mutation cohort in the microarray part of the study, but due to technical reasons, this result could not be confirmed by RT-qPCR. Therefore, we attempted to validate the result with the protein level. Immunohistochemical analysis of *NTRK3* expression may be difficult as its expression pattern in thyroid, particularly in MTC, is still not well understood. We consistently observed strong immunostaining in all MEN2B samples, present in both tumour C-cells and the surrounding follicular cells, while TrkC staining was negative in all but one analysed MEN2A-like tumour. However, we need to add that we observed non-specific staining in follicular cells. Positive staining by antibodies of both tumour and normal cells has also been reported by other authors who encountered similar obstacles; in many cases, the non-specific staining in normal cells was not possible to reduce^50^,^51^. Therefore, we consider the validation was successful, keeping in mind that most of the tumour samples were previously included in the microarray study. We also checked whether high expression of *NTRK3* gene was related to *ETV6/NTRK3* rearrangement, as reported in papillary thyroid cancer^52^,^53^, but we did not find the *ETV6/NTRK3* fusion gene in MTC samples. This is the first study showing the results of *ETV6/NTRK3* rearrangement in medullary thyroid cancer.

In our data set, none of the genes with FDR <0.05 proposed by Jain *et al*.^28^ were confirmed by comparison between MEN2A-like and MEN2B-like MTC samples; similarly, no differences were observed regarding to the genes reported by Watanabe *et al*.^29^ and Maliszewska *et al*.^31^.

Detailed analysis of a large MTC dataset supports the conclusion that differences in the gene expression of hereditary and sporadic MTC tumours are minor, if they really exist. This result enforces the hypothesis that hereditary and sporadic MTC activate the same genetic pathway regardless of *RET* mutation status.

Further, the difference in the tumour transcriptome between *RET* mutations initiating the MEN2A and MEN2B types of MTC is restricted to a few genes, which are related to cell proliferation, growth and differentiation. However, further investigations are necessary to demonstrate any clinical impact of these intriguing findings.

These results may also suggest that in MTC, not only does genetic predisposition play a significant role but epigenetic regulation could also be an important factor to understand the differences in the phenotypes of MTC subtypes. Importantly, our results indicate a rather homogeneous MTC gene expression profile.

## Methods

### Patient group

Tumour samples were obtained from 86 MTC patients (52 sporadic and 34 hereditary) (Supplementary Information Table S1) including 60 women and 26 men, with a mean age at diagnosis of 46 years. The proportions of females and males with sporadic MTC (67% and 33%, respectively) were similar to hereditary MTC (73% and 27%, respectively). All samples were obtained intraoperatively in the Maria Sklodowska-Curie Memorial Cancer Center, Gliwice Branch. The study was conducted after the approval of the Bioethics Committee MSC Memorial Cancer Center and Institute of Oncology, Gliwice Branch, was granted. All methods were performed in accordance with the relevant guidelines and regulations approved by the Bioethics Committee. Informed written consent was obtained from all patients or caregivers for the use of their tissues for analysis in this study.

### Tissue samples

Tumour tissue removed during surgical resection was snap-frozen on dry ice and stored at −80 °C until use. Total RNA was extracted by the Chomczynski-Sacchi method^54^ and purified using RNeasy Mini Kits (Qiagen GmbH, Hilden, Germany); its integrity (RIN value) was assessed using a Bioanalyzer 2100 (Agilent Technologies). The average RIN score for RNA samples used in these experiments was 7.13 (5.2–8.6).

### *RET* germline mutations

Genomic DNA was extracted from peripheral blood nucleated cells by the desalting method^55^. Mutation screening was performed according to a standard algorithm approved by the American and European MTC Management Guidelines^19^,^56^, which assumes the analysis of exons 10, 11, 13, 14, 15 and 16. These exons were sequenced directly using Big Dye 1.1 reagent and a 3130xl Genome Analyser (Life Technologies, Carlsbad, USA). We excluded exon 8 from analysis because a mutation at codon 533 of exon 8 is mainly related to a group of patients with Greek and Brazilian ancestry. To assess the possible presence of this type of *RET* gene mutation in a Polish population, we performed a study in which we screened exon 8 in 100 *RET* negative patients in codons 10,11 and 13–16, and we did not find any mutation in codon 8.

### *RET* somatic mutations

Genomic DNA was extracted from tissue samples by the DNeasy Blood and Tissue Kit (Qiagen GmbH, Hilden, Germany). All 21 exons of the *RET* gene were sequenced directly as described above. In patients with detected germline *RET* mutations, the somatic status was assumed to be identical, and the test was not repeated on tumour DNA.

### Datasets

Analyses were carried out on four data sets: two were analysed by microarrays (Sets A, B), one was analysed by RT-qPCR (Set C), and one was analysed by immunohistochemistry assay (Set D) (Supplementary Information Table S1)

### Microarray analysis Sets

Set A (Cancer Genetic Background Analysis Set) included 60 MTC samples to compare gene expression profiles between hereditary and sporadic MTC. Twenty-two were hereditary MTCs (including 2 families with 2 samples each), 19 were sporadic MTCs with somatic *RET* mutations, 15 were sporadic MTCs without somatic *RET* mutations, and 4 were sporadic MTC samples with unknown *RET* somatic mutation status (Supplementary Information Table S1). Array profiling was performed following the manufacturer’s protocol with minor modifications. Briefly, 250 ng of total RNA was processed as described in the GeneChip WT PLUS Reagent Kit Manual “Target Preparation for GeneChip Whole Transcript (WT) Expression Arrays, P/N 703174 Rev. 2” using only half the cDNA as template for the IVT reaction. cRNA was hybridized to the GeneChip Gene 1.0 ST arrays (Affymetrix). Washing, staining and scanning were performed according to the “GeneChip Expression Wash, Stain and Scan User Manual for Cartridge Arrays, P/N 702731 Rev. 3”.

Set B (Mutational Analysis Set) included 30 samples from Set A for comparison of gene expression profiles between tumours with MEN2A-like and MEN2B-like mutations. This set contained 21 MTC samples with MEN2A-like mutations (exons 10 and 11) and 9 MTC samples with MEN2B-like mutations (only exon 16), both germline and somatic.

### Quantitative Real-Time PCR Set

Set C (Validation Set, gene level) was used for the RT-qPCR validation of the differences of gene expression profile obtained by microarray analysis. It contained 25 Set A-independent MTC samples, including 2 samples of one family; (16 MEN2A-like and 9 MEN2B-like mutations; 13 sporadic and 12 hereditary MTCs) (Supplementary Information Table S1). Total RNA was extracted as described above. Reverse transcription was performed using Qiagen Omniscript RT Kits and 200 ng of input RNA in a final volume of 20 μl. All genes were amplified by the 7900HT Fast Real-Time PCR system (Life Technologies, Carlsbad, USA). The PCR reaction involved a first step at 50 °C (2 min; activation and incubation with AmpErase UNG) and 95 °C (10 min; activation of AmpliTaq Gold polymerase), followed by 40 cycles of amplification (95 °C, 15 s; 60 °C, 1 min). Each reaction contained 10 μl of TaqMan Universal Mastermix (Life Technologies, Carlsbad, USA), 200 nM of each primer, 2.8 μl of RNase-free water and 5 μl of diluted cDNA template. For amplicon design, we used the Roche Universal Probe Library, as well as the TaqMan MGB set for the *NTRK3* gene (Supplementary Information Table S2). The conditions for selecting the amplicons were as follows: 1) a sequence for all transcripts covered by the microarray probe, 2) an amplicon unique to the human transcriptome database (confirmed by BLAST), and 3) no known SNPs in any of the primer or probe sequences. A standard curve was generated for each amplicon using 8 concentrations in duplicates; a linear regression slope indicated amplification efficiency. For the normalization of the RT-qPCR data, we chose the *EIF3S10, HADHA* and *UBE2D2* genes. The normalization factor was obtained using the GeNorm applet for Microsoft Excel.

### Immunohistochemistry for TrkC

Set D (Validation Set, protein level) contained 12 MTC samples analysed in the microarray and RT-qPCR study (8 samples from Set A, 3 from Set C and 1 independent sample). In total, sections from 5 samples with MEN2A-like and 7 with MEN2B-like mutations were examined. To uncover antigens, tumour samples embedded by standard methods were deparaffinized and rehydrated with 3 in 1 Dako TRS High pH buffer, blocked in 3% H_2_O_2_, and washed with PBS. The samples were incubated with anti-human TrkC mouse monoclonal antibody (clone 75219, R&D Systems; 1:20 dilution). For visualization, we used the EnVision Flex+ system (Dako, K8002).

### Detection of ETV6-NTRK3 rearrangements

Tumour DNA was amplified using primers previously described by Leeman-Neill *et al*.^53^. In all, 24 DNA samples were available for analysis (Supplementary Information Table S1).

### Statistical analysis

#### Microarray data analysis

Raw microarray data from 60 MTC samples (Set A) were pre-processed by the RMA method using Affymetrix Expression Console software. The subsequent data analysis was performed in an R/Bioconductor environment. Control probe sets and low-variability probe sets (less than 15% of samples with at least 1.5-fold change in either direction from the median) were filtered out. Next, unsupervised analysis by hierarchical clustering was carried out, using the Ward agglomeration method operated on Euclidean distance measures. The selection of probe sets, which significantly distinguished between the analysed groups, was performed using the Welch t-test. The Benjamini-Hochberg false discovery rate (FDR) was used to assess the multiple testing errors. Sample classification was performed using support vector machine (svm) methods with a linear kernel, and the performance of the classifier was estimated by leave-one-out cross-validation (LOOCV). A gene was included in the classifier in each cross-validation step when it satisfied the significance criteria of a Welch t-test p value lower than 0.001 and a 4-fold change (up or down-regulated).

#### Real Time quantitative PCR data analysis

Normalized RT-qPCR data were assessed by the non-parametric Mann-Whitney U test adjusted by Bonferroni correction.

## Additional Information

**How to cite this article:** Oczko-Wojciechowska, M. *et al*. Differences in the transcriptome of medullary thyroid cancer regarding the status and type of *RET* gene mutations. *Sci. Rep.* **7**, 42074; doi: 10.1038/srep42074 (2017).

**Publisher's note:** Springer Nature remains neutral with regard to jurisdictional claims in published maps and institutional affiliations.

## Supplementary Material

Supplementary Information

## Figures and Tables

**Table 1:** Genes differentiating between hereditary and sporadic MTC with a FDR <0.05 (Set A).

Symbol	ProbeSet	Name	Fold-change	p-value	FDR	p-value validation set HG-U133A
*PTPRK*	8129418	protein tyrosine phosphatase, receptor type, K	0.48	0,0000255	0,0308	0,2
*KLF12*	7972003	Kruppel-like factor 12	0.69	0,0000585	0,0353	0,3
*CCL18*	8006594	chemokine (C-C motif) ligand 18 (pulmonary and activation-regulated)	2.88	0,0000412	0,0351	0,4
*SLC22A3*	8123246	solute carrier family 22 (extraneuronal monoamine transporter), member 3	2.12	0,0000696	0,0387	0,4
*FHOD3*	8020973	formin homology 2 domain containing 3	1.65	0,0000543	0,0353	0,6
*TAC3*	7964303	tachykinin 3	1.44	0,0000154	0,0223	0,7
*ZNF432*	8038942	zinc finger protein 432	0.74	0,0000536	0,0353	0,8
*LECT1*	7971838	leukocyte cell derived chemotaxin 1	3.3	0,0000976	0,0471	0,9
*TCERG1L*	7937059	transcription elongation regulator 1-like	1.4	0,0000019	0,0123	not available on HG-U133A
*DPP10*	8044700	dipeptidyl-peptidase 10 (non-functional)	2.85	0,0000034	0,0123	not available on HG-U133A
*NALCN*	7972601	sodium leak channel, non-selective	0.4	0,0000114	0,0223	not available on HG-U133A
*APOA1BP*	7906185	apolipoprotein A-I binding protein	1.46	0,0000143	0,0223	not available on HG-U133A
*ZNF404*	8037430	zinc finger protein 404	0.72	0,0000419	0,0351	not available on HG-U133A
*SPTSSB*	8091799	serine palmitoyltransferase, small subunit B	0.35	0,0000437	0,0351	not available on HG-U133A
*KIAA1737*	7975926	KIAA1737	0.76	0,0000922	0,0471	not available on HG-U133A

**Table 2:** Comparison of gene expression in samples with MEN2A-like and MEN2B-like *RET* mutations using probe sets with a FDR <0.05.

Symbol	ProbeSet	Mean expression in mutations		Fold-change	Uncorrected p-value	FDR
		MEN 2A-like	MEN 2B-like			
NTRK3*	7991186	27.86	134.47	0.21	2.70E-06	0.0195
*NALCN*	7972601	33.13	105.66	0.31	7.10E-06	0.0257
*ZC3H12C*	7943715	88.65	52.91	1.68	1.22E-05	0.0294
*HMGA2*	7964736	23.13	41.51	0.56	1.69E-05	0.0306
*HLA-DRB5*	8125436	52.45	147.6	0.36	2.38E-05	0.0344
*PDGFRL*	8144802	215.63	135.82	1.59	4.21E-05	0.0393
*CDC14B*	8162610	57.87	35.18	1.65	4.29E-05	0.0393
NNAT*	8062395	291.36	44.53	6.54	4.59E-05	0.0393
*ZNF658*	8161346	41.25	28.16	1.46	4.91E-05	0.0393
*HSD17B14*	8038213	129.36	78.49	1.65	5.43E-05	0.0393

**Table 3:** Genes used in classification by type of *RET* mutation.

*p* value	FDR	mean MEN2A-like mutations	mean MEN2B-like mutations	Fold-change	ProbeSet	Symbol
2.70E-06	0.0195	27.86	134.47	0.21	7991186	NTRK3
4.59E-05	0.0393	291.36	44.53	6.54	8062395	*NNAT*
0.000261	0.0957	108.38	597.72	0.18	8128087	GABRR1*
0.000951	0.127	263.98	65.09	4.06	8066347	PTPRT*

**Table 4:** Performance of the 4-gene classifier for prediction of MEN2A-like or MEN2B-like mutations (see Table 3).

Accuracy	Sensitivity	Specificity	PPV	NPV
90%	91%	87.50%	95%	78%
