# Corrigendum: Differences in the transcriptome of medullary thyroid cancer regarding the status and type of *RET* gene mutations

**DOI:** 10.1038/srep44347

**Published:** 2017-03-15

**Authors:** Malgorzata Oczko-Wojciechowska, Michal Swierniak, Jolanta Krajewska, Malgorzata Kowalska, Monika Kowal, Tomasz Stokowy, Bartosz Wojtas, Dagmara Rusinek, Agnieszka Pawlaczek, Agnieszka Czarniecka, Sylwia Szpak-Ulczok, Tomasz Gawlik, Ewa Chmielik, Tomasz Tyszkiewicz, Barbara Nikiel, Dariusz Lange, Michal Jarzab, Malgorzata Wiench, Barbara Jarzab

Scientific Reports 7: Article number: 4207410.1038/srep42074; published online: 02 09 2017; updated: 03 15 2017

In this Article, an affiliation for Bartosz Wojtas was omitted. The correct affiliations for Bartosz Wojtas are listed below:

Department of Nuclear Medicine and Endocrine Oncology, Maria Sklodowska-Curie Memorial Cancer Center and Institute of Oncology, Gliwice Branch, Poland.

Laboratory Molecular Neurobiology, The Nencki Institute of Experimental Biology of the Polish Academy of Sciences, Poland.

