# Supplementary material for: Differences in the transcriptome of medullary thyroid cancer regarding the status and type of RET gene mutations
Source: Sci Rep. 2017 Feb 9;7:42074. doi: 10.1038/srep42074 (PMC5299608; doi:10.1038/srep42074)
Supplement: Supplementary Information [file srep42074-s1.pdf]

Differences in the transcriptome of medullary thyroid cancer regarding the status and type of *RET* gene mutations

Malgorzata Oczko-Wojciechowska[1]*^, Michal Swierniak[1,2]^, Jolanta Krajewska[1]^, Malgorzata Kowalska[1], Monika Kowal[1], Tomasz Stokowy[3], Bartosz Wojtaś[1], Dagmara Rusinek[1], Agnieszka Pawlaczek[1], Agnieszka Czarniecka[4], Sylwia Szpak-Ulczok[1], Tomasz Gawlik[1], Ewa Chmielik[5], Tomasz Tyszkiewicz[1], Barbara Nikiel[5], Dariusz Lange[5], Michal Jarzab[6], Malgorzata Wiench[7], Barbara Jarzab[1]

^ These three authors contributed equally to this study

[1] Department of Nuclear Medicine and Endocrine Oncology, Maria Sklodowska-Curie Memorial Cancer Center and Institute of Oncology, Gliwice Branch, Poland

[2] Genomic Medicine, Medical University of Warsaw, Poland

[3] Department of Clinical Science, University of Bergen, Norway

[4] The Oncology and Reconstructive Surgery Clinic, Maria Sklodowska-Curie Memorial Cancer Center and Institute of Oncology, Gliwice Branch, Poland

[5] Tumour Pathology Department, Maria Sklodowska-Curie Memorial Cancer Center and Institute of Oncology, Gliwice Branch, Poland

[6] III Radiotherapy Clinic, Maria Sklodowska-Curie Memorial Cancer Center and Institute of Oncology, Gliwice Branch, Poland

[7] College of Medical and Dental Sciences, University of Birmingham, United Kingdom

Supplementary Information

Supplementary Table 1. All samples used in analysis

No	Sample code	MTC	Gender	*RET* germline mutation	*RET* somatic mutation	Group hereditary vs sporadic	Group MEN2A vs MEN2B-like	Method of analysis	ETV6-NTRK3 analysisi	SET	ICH
1	MTC 34	hereditary	F	p.V804L GTG/TTG	NA	hereditary		Human Gene 1.0 ST	NA	A	NA
2	MTC 57	hereditary	F	p.G768D GAG/GAT	NA	hereditary		Human Gene 1.0 ST	NA	A	NA
3	MTC 20	sporadic	F	0	0	sporadic		Human Gene 1.0 ST	NA	A	NA
4	MTC 22	sporadic	F	0	0	sporadic		Human Gene 1.0 ST	NA	A	NA
5	MTC 10	sporadic	M	0	NA	sporadic		Human Gene 1.0 ST	NA	A	NA
6	MTC 100	sporadic	M	0	del (7 bp) exon 11	sporadic		Human Gene 1.0 ST	NA	A	NA
7	MTC 110	sporadic	F	0	p.M918T ATG/ACG	sporadic		Human Gene 1.0 ST	NA	A	NA
8	MTC 111	sporadic	F	0	0	sporadic		Human Gene 1.0 ST	NA	A	NA
9	MTC 118	sporadic	F	0	0	sporadic		Human Gene 1.0 ST	NA	A	NA
10	MTC 119	sporadic	M	0	0	sporadic		Human Gene 1.0 ST	NA	A	NA
11	MTC 122	sporadic	F	0	0	sporadic		Human Gene 1.0 ST	NA	A	NA
12	MTC 123	sporadic	M	0	del/ins exon 11	sporadic		Human Gene 1.0 ST	NA	A	NA
13	MTC 124	sporadic	F	0	0	sporadic		Human Gene 1.0 ST	NA	A	NA
14	MTC 128	sporadic	F	0	0	sporadic		Human Gene 1.0 ST	NA	A	NA
15	MTC 132	sporadic	F	0	0	sporadic		Human Gene 1.0 ST	ND	A	NA
16	MTC 133	sporadic	F	0	0	sporadic		Human Gene 1.0 ST	NA	A	NA

17	MTC 136	sporadic	F	0	0	sporadic		Human Gene 1.0 ST	NA	A	NA
18	MTC 14	sporadic	F	0	del (6 bp) exon 11	sporadic		Human Gene 1.0 ST	NA	A	NA
19	MTC 140	sporadic	F	0	0	sporadic		Human Gene 1.0 ST	NA	A	NA
20	MTC 143	sporadic	F	0	0	sporadic		Human Gene 1.0 ST	NA	A	NA
21	MTC 4	sporadic	M	0	NA	sporadic		Human Gene 1.0 ST	NA	A	NA
22	MTC 89	sporadic	F	0	p.A883F GCT/TTT	sporadic		Human Gene 1.0 ST	NA	A	NA
23	MTC 24	sporadic	M	0	p.H568N CAC/AAC	sporadic		Human Gene 1.0 ST	NA	A	NA
24	MTC 73	sporadic	F	0	del (19 bp) exon 11	sporadic		Human Gene 1.0 ST	NA	A	NA
25	MTC 87	sporadic	F	0	0	sporadic		Human Gene 1.0 ST	NA	A	NA
26	NIS 622	sporadic	F	0	0	sporadic		Human Gene 1.0 ST	NA	A	NA
27	NIS 638	sporadic	F	0	NA	sporadic		Human Gene 1.0 ST	NA	A	NA
28	NIS 683	sporadic	F	0	NA	sporadic		Human Gene 1.0 ST	NA	A	NA
29	MTC 104	hereditary	F	p.L790F TAT/TTT	NA	hereditary		Human Gene 1.0 ST	NA	A	NA
30	MTC 154	hereditary	M	p.L790F TTG/TTT	NA	hereditary		Human Gene 1.0 ST	NA	A	NA
31	MTC 155	hereditary	M	p.C634G TGC/GGC	NA	hereditary	MEN2A-like	Human Gene 1.0 ST	NA	A, B	NA
32	MTC 16	hereditary	M	p.C620Y TGC/TAC	NA	hereditary	MEN2A-like	Human Gene 1.0 ST	NA	A, B	NA
33	MTC 32	hereditary	F	p.C618W TGC/TGG	NA	hereditary	MEN2A-like	Human Gene 1.0 ST	NA	A, B	E
34	MTC 38	hereditary	F	p.C634W TGC/TGG	NA	hereditary	MEN2A-like	Human Gene 1.0 ST	NA	A, B	NA
35	MTC 63	hereditary	M	p.C620Y TGC/TAC	NA	hereditary	MEN2A-like	Human Gene 1.0 ST	NA	A, B	NA
36	MTC 75	hereditary	F	p.C618W TGC/TGG	NA	hereditary	MEN2A-like	Human Gene 1.0 ST	NA	A, B	NA
37	MTC 82	hereditary	F	p.C618S TGC/AGC	NA	hereditary	MEN2A-like	Human Gene 1.0 ST	NA	A, B	NA

38	NIS 258	hereditary	F	p.C634G TGC/GGC	NA	hereditary	MEN2A-like	Human Gene 1.0 ST	NA	A, B	E
39	NIS 262	hereditary	F	p.C634G TGC/GGC	NA	hereditary	MEN2A-like	Human Gene 1.0 ST	NA	A, B	NA
40	NIS 634	hereditary	F	p.C634W TGC/TGG	NA	hereditary	MEN2A-like	Human Gene 1.0 ST	NA	A, B	NA
41	NIS 657	hereditary	F	p.C620Y TGC/TAC	NA	hereditary	MEN2A-like	Human Gene 1.0 ST	NA	A, B	NA
42	NIS 659	hereditary	F	p.C634W TGC/TGG	NA	hereditary	MEN2A-like	Human Gene 1.0 ST	NA	A, B	NA
43	NIS 661	hereditary	F	p.M918T ATG/ACG	NA	hereditary	MEN2B-like	Human Gene 1.0 ST	NA	A, B	NA
44	MTC 137	sporadic	M	0	p.C630G TGC/GGC	sporadic	MEN2A-like	Human Gene 1.0 ST	NA	A, B	NA
45	MTC 40	sporadic	F	0	p.C634R TGC/CGC	sporadic	MEN2A-like	Human Gene 1.0 ST	NA	A, B	NA
46	MTC 45	sporadic	M	0	p.C630R TGC/CGC	sporadic	MEN2A-like	Human Gene 1.0 ST	NA	A, B	NA
47	MTC 54	sporadic	F	0	p.M918T ATG/ACG	sporadic	MEN2B-like	Human Gene 1.0 ST	ND	A, B	NA
48	MTC 65	sporadic	F	0	p.C634W TGC/TGG	sporadic	MEN2A-like	Human Gene 1.0 ST	NA	A, B	NA
49	MTC 85	sporadic	F	0	p.C634R TGC/CGC	sporadic	MEN2A-like	Human Gene 1.0 ST	NA	A, B	E
50	MTC 55	sporadic	M	0	p.M918T ATG/ACG	sporadic	MEN2B-like	Human Gene 1.0 ST	NA	A, B	E
51	MTC 109	hereditary	F	p.C634G TGC/GGC	NA	hereditary	MEN2A-like	Human Gene 1.0 ST	NA	A, B	E
52	MTC 12	hereditary	M	p.C618W TGC/TGG	NA	hereditary	MEN2A-like	Human Gene 1.0 ST	NA	A, B	NA
53	MTC 153	hereditary	F	p.M918T ATG/ACG	NA	hereditary	MEN2B-like	Human Gene 1.0 ST	ND	A, B, D	E
54	MTC 30	hereditary	F	p.C634R TGC/CGC	NA	hereditary	MEN2A-like	Human Gene 1.0 ST	NA	A, B, D	NA
55	MTC 8	hereditary	F	p.C634W TGC/TGG	NA	hereditary	MEN2A-like	Human Gene 1.0 ST	NA	A, B, D	NA
56	MTC 120	sporadic	F	0	p.M918T ATG/ACG	sporadic	MEN2B-like	Human Gene 1.0 ST	ND	A, B, D	NA
57	MTC 36	sporadic	F	0	p.M918T ATG/ACG	sporadic	MEN2B-like	Human Gene 1.0 ST	ND	A, B, D	E
58	MTC 42	sporadic	F	0	p.M918T ATG/ACG	sporadic	MEN2B-like	Human Gene 1.0 ST	ND	A, B, D	NA

59	MTC 98	sporadic	F	0	p.M918T ATG/ACG	sporadic	MEN2B-like	Human Gene 1.0 ST	ND	A, B, D	NA
60	MTC 91	sporadic	F	0	p.M918T ATG/ACG	sporadic	MEN2B-like	Human Gene 1.0 ST	ND	A, B, D	E
61	MTC 117	sporadic	F	0	p.C634R TGC/CGC	sporadic	MEN2A-like	qRT-PCR	ND	C	NA
62	MTC 28	sporadic	M	0	p.C634R TGC/CGC	sporadic	MEN2A-like	qRT-PCR	ND	C	NA
63	MTC 113	sporadic	M	0	p.M918T ATG/ACG	sporadic	MEN2B-like	qRT-PCR	ND	C	NA
64	MTC 134	sporadic	M	0	p.M918T ATG/ACG	sporadic	MEN2B-like	qRT-PCR	ND	C	NA
65	MTC 144	sporadic	M	0	p.M918T ATG/ACG	sporadic	MEN2B-like	qRT-PCR	ND	C	NA
66	MTC 84	sporadic	F	0	p.M918T ATG/ACG	sporadic	MEN2B-like	qRT-PCR	ND	C	NA
67	MTC 131	sporadic	F	0	p.C634R TGC/CGC	sporadic	MEN2A-like	qRT-PCR	ND	C	NA
68	MTC 138	sporadic	F	0	p.C634R TGC/CGC	sporadic	MEN2A-like	qRT-PCR	ND	C	NA
69	MTC 125	sporadic	M	0	p.M918T ATG/ACG	sporadic	MEN2B-like	qRT-PCR	ND	C	NA
70	MTC 126	sporadic	F	0	p.M918T ATG/ACG	sporadic	MEN2B-like	qRT-PCR	ND	C	NA
71	MTC 145	sporadic	M	0	p.M918T ATG/ACG	sporadic	MEN2B-like	qRT-PCR	ND	C	NA
72	MTC 69	sporadic	M	0	p.M918T ATG/ACG	sporadic	MEN2B-like	qRT-PCR	ND	C	NA
73	NIS 478	hereditary	M	p.C620Y TGC/TAC	NA	hereditary	MEN2A-like	qRT-PCR	NA	C	NA
74	NIS 276	hereditary	F	p.C620A TGC/CGC	NA	hereditary	MEN2A-like	qRT-PCR	NA	C	E
75	NIS 303	hereditary	F	p.C634R TGC/CGC	NA	hereditary	MEN2A-like	qRT-PCR	NA	C	NA
76	NIS 388	hereditary	M	p.C634G TGC/GGC	NA	hereditary	MEN2A-like	qRT-PCR	NA	C	NA
77	MTC 6	hereditary	F	p.C620Y TGC/TAC	NA	hereditary	MEN2A-like	qRT-PCR	ND	C	E
78	MTC 61	hereditary	F	p.C634R TGC/CGC	NA	hereditary	MEN2A-like	qRT-PCR	ND	C	NA
79	NIS 474	hereditary	F	p.C634G TGC/GGC	NA	hereditary	MEN2A-like	qRT-PCR	NA	C	NA

80	NIS 507	hereditary	M	p.C634R TGC/CGC	NA	hereditary	MEN2A-like	qRT-PCR	NA	C	E
81	NIS 630	hereditary	F	p.C634G TGC/GGC	NA	hereditary	MEN2A-like	qRT-PCR	NA	C	NA
82	NIS 278	hereditary	M	p.C620A TGC/GCG	NA	hereditary	MEN2A-like	qRT-PCR	NA	C	NA
83	MTC 59	sporadic	F	0	p.C634F TGC/TTC	sporadic	MEN2A-like	qRT-PCR	NA	C,D	NA
84	MTC 142	sporadic	M	0	p.M918T ATG/ACG	sporadic	MEN2B-like	qRT-PCR	ND	C,D	NA
85	MTC 156	hereditary	F	p.C634R TGC/CGC	NA	hereditary	MEN2A-like	qRT-PCR	ND	C,D	NA
86	NIS476	hereditary	F	p.V804L GTG/TTG	NA	hereditary	MEN2A-like	NA	NA	D	E

NA - not analyzed

ND - not detected

ICH - immunohistochemistry

Set A: Cancer Genetic Background Analysis; microarray analysis; Hereditary vs Sporadic MTC

Set B: Mutational Analysis; MEN2 like vs MEN2B like

Set C: Validation Set for QPCR Aanlysis

Set D: Validation Set, protein level (ICH)

Supplementary Table 2. Sequences of primers and numbers of probes used for RT-qPCR validation part of study

	Roche Universal Probe Library		
Gene	Probe	Forward Primer	Reverse Primer
APOA1BP	77	aaatggacatccctttccttg	gaagctgaagccaaagatgg
CDC14B	66	gggtgccattgcagtacatt	agatcctgacccacgcaat
FHOD3	27	aggccaggttggaaaggt	aggccaggttggaaaggt
HMGA2	38	gcgcctcagaagagaggac	cttcccctgggtctcttagg
HSD17B14	49	ccgcctggattgtgttgt	ggtctcctcaggcctctgt
KIAA1337	33	aagaatgtgcttgtcaaacagg	cttcaaaggagggctggac
KLF12	2	gggactctgtgtgagggaaa	tgttcttagagtcacattgatcctg
NALCN	17	acaacagcatgcaacctgag	ttcctcctcgatccgaca
NNAT	65	gtacatcttccgcgtgctg	cttctcgcaatgggctgt
NTRK3	21	ccgtacgagagggtgacaat	tggtccagttcagattggtct
TCERG1L	83	tccccacggtggtgttag	gctggtcaccttaaggctgttat
ZC3H12C	13	ttcaaatgccattgtaaaaactactc	tctgtggctgttcttatgctct
ZNF 404	63	tcagcgatgttgccatagac	gctttcagttgtgaaattaaagtcc
ZNF 432	17	ggagttcacctgggaggagt	aacatcacatcccggtacaaa
ZNF 658	85	gagcacaaggtcaaggaagc	gcaacaaaaacaaaaccctgat
	TaqMan gene expression assay		
HMGA2	Hs00971725_m1		
NTRK3	Hs00176797_m1		
PDGFRL	HS00185122_m1		

Supplementary Fig. S1. Immunohistochemical staining of TrkC receptor in MEN2A-like (black coloured) and MEN2B-like (blue coloured) samples. All MEN2B-like samples were strongly positive for TrkC, while MEN2A-like were negative except for one tumour which exhibited strong TrkC staining (MTC59 sample)

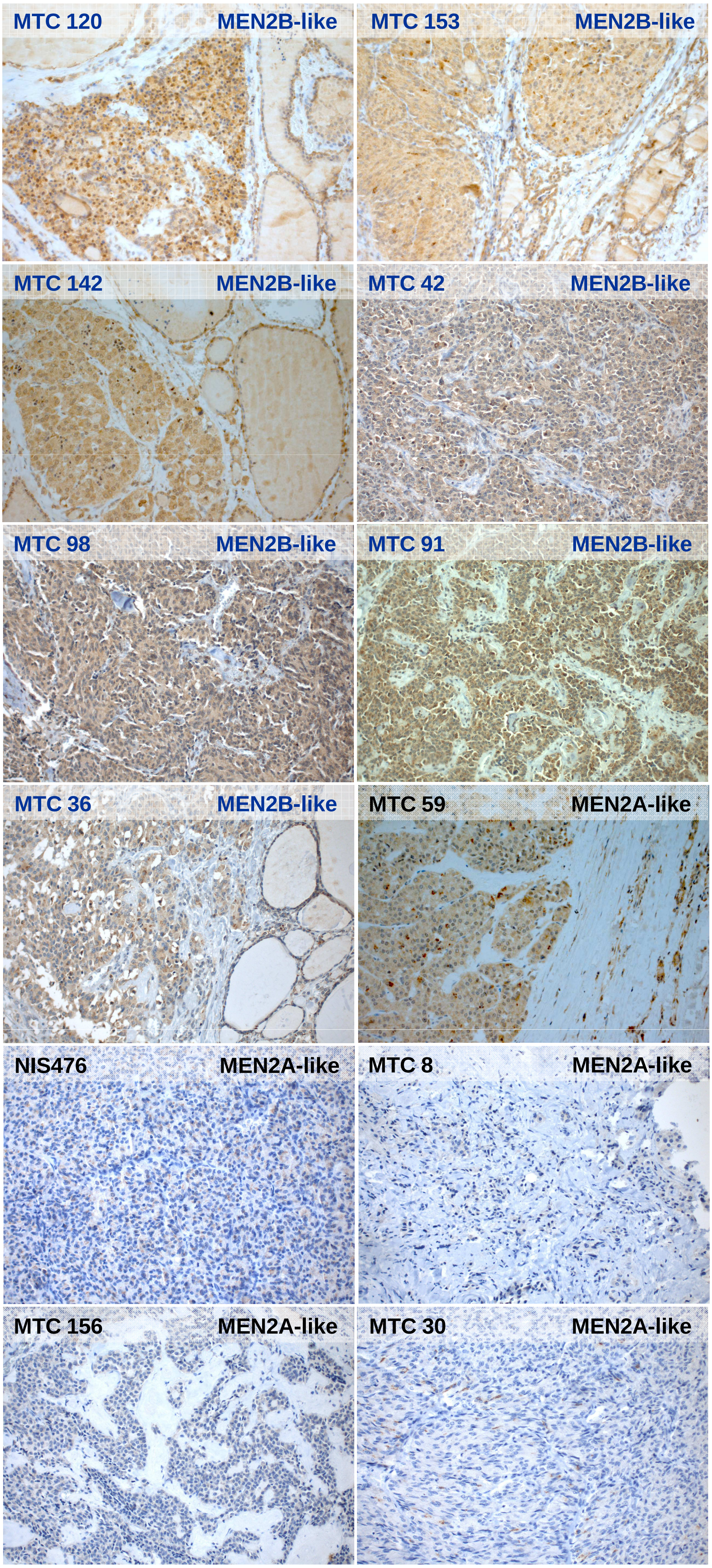

Supplementary Fig. S2. Result of analysis ETV-6-NTRK3 rearrangements. All 24 MTC samples were negative using PCR method.

006 028 036 042 044 054 061 069 084 091 098 113 117 120 125 126 M

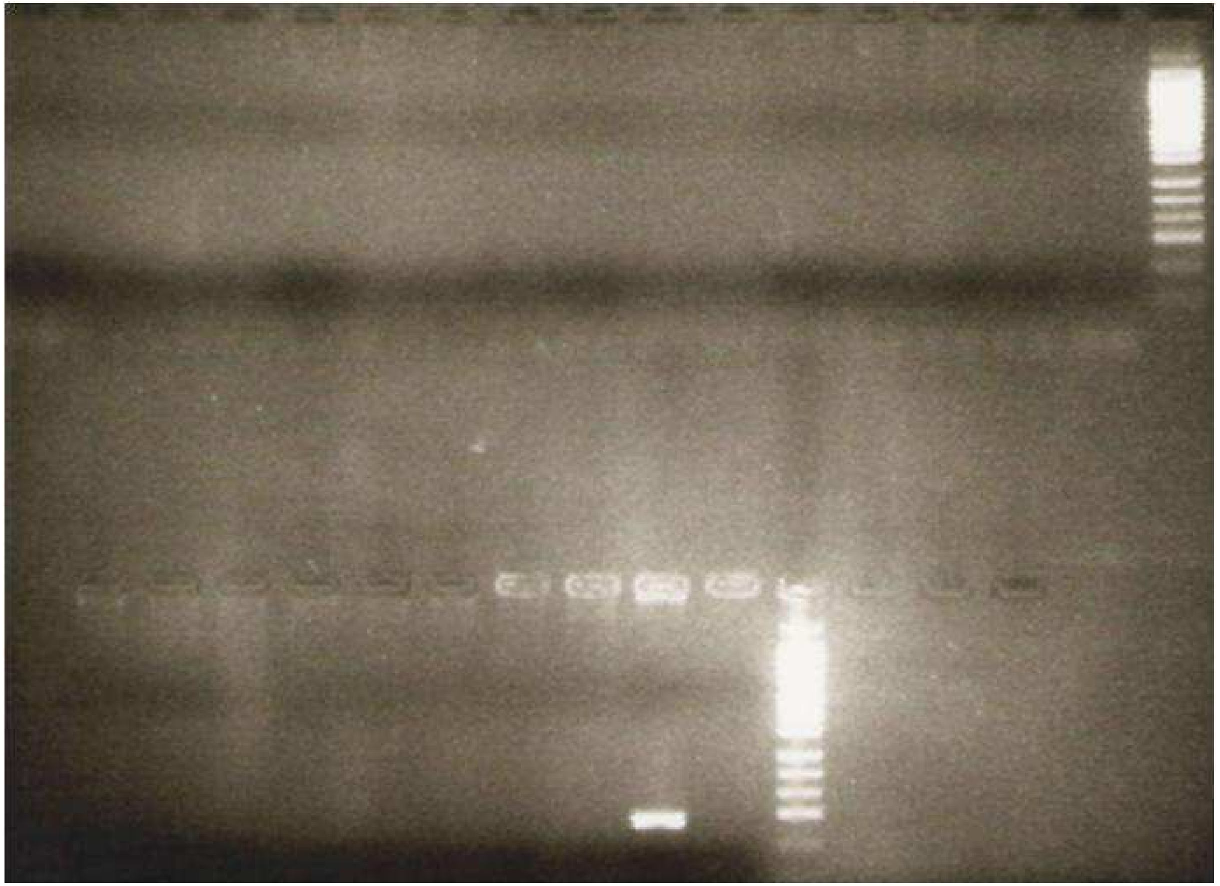

131 132 138 142 144 145 153 156 K+ K- M

K+ positive control
K- negative control
M – DNA size marker
